# Analysis of Selected Properties of Biocomposites Based on Polyethylene with a Natural Origin Filler

**DOI:** 10.3390/ma13184182

**Published:** 2020-09-20

**Authors:** Emil Sasimowski, Łukasz Majewski, Marta Grochowicz

**Affiliations:** 1 Department of Technology and Polymer Processing, Faculty of Mechanical Engineering, Lublin University of Technology, 36 Nadbystrzycka Street, 20-618 Lublin, Poland; e.sasimowski@pollub.pl; 2 Department of Polymer Chemistry, Institute of Chemical Sciences, Faculty of Chemistry, Maria Curie-Sklodowska University in Lublin, 33 Gliniana Street, 20-614 Lublin, Poland; mgrochowicz@poczta.umcs.lublin.pl

**Keywords:** injection moulding, biomass waste, wheat bran, mechanical properties, thermal properties, agricultural waste, lignocellulosic materials, polymer processing

## Abstract

The study investigates the effect of the content and size of wheat bran grains on selected properties of a lignocellulosic biocomposite on a polyethylene matrix. The biocomposite samples were made by injection method of low-density polyethylene with 5%, 10% and 15% by weight of wheat bran. Three bran fractions with grain sizes <0.4 mm, 0.4–0.6 mm and 0.6–0.8 mm were used. The properties of the mouldings (after primary shrinkage) were examined after their 2.5-year natural aging period. Processing properties, such as MFR (mass flow rate) and processing shrinkage, were determined. Selected physical, mechanical and structural properties of the produced biocomposite samples were tested. The results allowed the determination of the influence of both the content of bran and the size of its grains on such properties of the biocomposite as: color, gloss, processing shrinkage, tensile strength, MFR mass flow rate, chemical structure (FTIR), thermal properties (DSC, TG), p-v-T relationship. The tests did not show any deterioration of the mechanical characteristics of the tested composites after natural aging.

## 1. Introduction

The use of composite polymer materials is now a common practice that allows adaptation of material properties to even most demanding requirements of the automotive, electrical, aviation and household industries [1,2]. Modifications of properties are most often made by mixing polymers with various types of fillers or fibers [3]. The most popular in mass applications are mineral fillers such as mica, talc, kaolin, wollastonite, calcium carbonate, silica and montmorillonite [4,5,6]. There are many reasons for the high popularity of mineral fillers. These include relatively low price, availability, a large range of sizes from micro to nano and a predictable and reproducible effect on the properties of the final product. The main advantages of their use are higher stiffness and the reduction in processing shrinkage and flammability—the properties that were a significant limitation for the use of unmodified thermoplastic polymers [1,7]. However, due to growing ecological awareness and the increasing level of environmental pollution, an increased interest in biocomposites based on biodegradable polymers and containing fillers of natural origin has been observed for several years [8,9,10]. The practical use of biodegradable polymers, such as polylactide or thermoplastic starch, is still limited. The reason is the rigorous and technically difficult process of their production and processing, which results in a much higher price than that of petrochemical plastics [11,12,13]. Therefore, an intensively developed trend in the field of polymer processing is the use of natural fillers, which, in addition to their unique properties, allows the reduction in the consumption of polymer materials. This gives measurable benefits both in the processing of biopolymers, by significantly reducing the price of the composite and allowing for their wider use, and petrochemical polymers, by reducing environmental pollution [14,15,16,17].

All kinds of natural fillers used in polymer composite production are called lignocellulosic materials due to their chemical structure [18]. They contain mainly cellulose, hemicellulose and lignin as well as small amounts of fats, proteins and other substances, which is directly related to the structure of plant cells [19,20]. The individual components differ from each other in terms of both their structure and chemical composition, and therefore, they have different effects on the functional and processing properties of composite materials [21]. The three main components of lignocellulosic materials differ significantly in their thermal stability. Cellulose has the highest decomposition temperature, followed by lignin, whereas hemicellulose has the lowest heat resistance [22]. Thus, from the point of view of processing polymer composites, it is advantageous to choose fillers with a predominant amount of cellulose, as they will show better thermal stability [21,22,23]. However, cellulose chains can create both crystalline and amorphous structures, which differ in properties [18,24]. As a rule, amorphous cellulose, similar to hemicellulose, is characterized by a more intensive absorption of moisture and a lower decomposition temperature, which in turn makes it more susceptible to degradation [25]. Moreover, it has been proved that the share of individual lignocellulose components significantly influences its mechanical characteristics and, consequently, the properties of composite materials [26,27].

The strong relationship between the properties and the chemical composition triggered an intensive search for new natural fillers, which, apart from reducing production costs and limiting the consumption of polymer materials, will enable the production of biocomposites with the desired properties [28]. Initially, wood dust dominated in the production of polymer biocomposites with natural fillers, but despite the possibility of a high degree of filling, the price of polymer-wood composites is still relatively high [29,30]. This led to the emergence of a strongly developing trend of producing lignocellulosic biocomposites with the use of various types of natural waste from agriculture, which allows for solving some of the problems related to their disposal [13,31,32]. Currently, technological wastes from wood, agricultural and food industries are considered a valuable source of natural fillers in the production of polymer composites with unique properties [33]. According to the literature data, the lignocellulosic materials used as fillers are classified according to their origin and botanical functions, including bast, leaves, seeds, fruit, wood, stems and grasses [33,34]. Additionally, technological waste from the food industry can also be used, for example, crushed walnut shells [35,36], hazelnut [37] and peanut [36], coffee grounds [26], rice bran [36,38] and wheat [30,39] or sugar cane bagasse [40].

The use of biofillers obtained from natural waste provides obvious benefits, such as reducing the consumption of petrochemical polymers [41], rapid degradation in environmental conditions [42,43], lower price [44], lower density [45], reduction in tribological wear of processing tools [45,46] and higher availability [47] compared to mineral fillers. It also happens that the use of specific types of biofillers provides unique properties, such as increasing the dielectric strength [48] or reducing oxygen permeability [49]. The most commonly used methods of processing polymer composites with a natural filler are extrusion and injection. By extrusion, both finished products from lignocellulosic composites and composite granules, which are the input material for subsequent processing, are produced [45,50]. The use of lignocellulosic fillers causes a number of limitations that hinder the processing of composites with the above-mentioned methods. The most important are low thermal stability and hygroscopicity [51,52].

Thermal stability determines the allowable processing temperature, as the thermal decomposition of the filler has a negative impact on the mechanical and visual properties of the finished products. Therefore, biofillers are not suitable for the production of composites based on polymers with a high melting point [53]. One should also take into account autothermal phenomena caused by intense shearing in the plasticizing systems of extruders and injection moulding machines, which increase the temperature of the material above the set temperature [54]. Therefore, the most commonly used petrochemical material for the production of biocomposites is polyethylene, due to its very good processing characteristics and the possibility of effective processing at low temperatures, even below 150 °C [55,56]. It is related to the temperature of hemicellulose decomposition, which runs in the range of 150–350 °C [57]. On the other hand, from the group of biodegradable polymers, the most frequently used is polylactic acid (PLA) [58], which requires appropriate preparation or selection of the filler due to the higher processing temperature. The literature describes methods of increasing the thermal stability of lignocellulose even up to 260 °C by separating the least thermally resistant components and using acid hydrolysis to remove amorphous cellulose [23,59], but these are complex procedures that eliminate the economic aspect of using cheap natural fillers.

The hydrophilic nature and the ability to intensively absorb moisture constitute further difficulties in the design and production of biocomposites. Hydrophilicity limits the force of interactions at the interface when filling hydrophobic materials [60,61]. This leads to difficulties in the proper distribution of the filler in the matrix and the formation of agglomerates, deterioration of mechanical properties and lack of repeatability in the final products [62,63]. The solution may be the use of compatibilizers and chemical modifications of the filler [64,65], but these adversely affect the economy of production and are not always proecological solutions [66,67,68]. In the case of most biodegradable polymers, the problem of polarity does not occur, but most of these plastics are highly hygroscopic, similar to lignocellulosic fillers [69]. The moisture content makes processing much more difficult, leading to the formation of pores, surface collapses and dimensional deviations, an increase in pressure in the plasticizing systems of processing machines, and may accelerate the wear of their working elements [18,52]. Research shows that full soaking of biocomposites with a natural filler based on polyethylene, when fully immersed in water, may take up to several dozen hours [70]. Thus, removing the moisture found in the structure of such a composite requires a sufficiently long drying procedure. Composite granules produced by extrusion and intended for further processing require storage in special bags, processing immediately after opening, the use of containers with a drying function and plasticizing systems with degassing, what may constitute additional difficulties [71,72].

From the processing point of view, several other disadvantages of lignocellulosic fillers can be listed. One of them is the deterioration of the processability in relation to unfilled plastic [70], caused by the increase in viscosity along with the increase in the filler content [73,74]. This results in a reduction in the efficiency of the process and an increased load on drive systems or the need to use additives facilitating processing [50,70]. Moreover, biofillers are characterized by variations in their chemical composition and structure, depending on geographic and climatic factors, which in turn causes variability in the mechanical and processing characteristics of biocomposites [13,33]. The quality and composition of biofillers depend on the exposure of the lignocellulosic material to many factors, which can be divided into four stages: plant growth, harvesting stage, obtaining the filler, transport and storage [45,75]. An additional effect of the use of biofillers is their influence on the look of the final product, its color and surface structure [70]. Sometimes this may be a desired characteristic effect, but otherwise, it will require the use of dyes or pigments [55].

The production of polymer biocomposites with lignocellulosic fillers has many advantages, but at the same time is technically difficult and requires the special design of both the composition and processing. Ecological considerations support the idea of the constant search for new natural fillers using the available natural waste resources. On the other hand, the study of processing aspects allows the determination of the usefulness of new fillers and the development of a technology for producing biocomposites with their use. Therefore, the aim of the presented study was to investigate selected properties of biocomposites based on low-density polyethylene with the addition of wheat bran. The presented research included the determination of the processing characteristics of the tested biocomposites by injection method, determination of the influence of wheat bran on the thermal properties and visual features of the tested composition, and the assessment of the influence of bran on the mechanical characteristics of the composition over long periods of time.

## 2. Experimental Conditions 

### 2.1. Test Stand

Measurement samples in the form of spatulas were made using a CS-88/63 screw injection moulding machine (Vihorlat Snina n.p., Snina, Słowacja), equipped with a mould with two cavities, shaped and sized according to the EN ISO 294-1: 2017-07 [76] standard with pinpoint gates. The injection process was carried out in accordance with the technological parameters presented in Table 1 by introducing a premixed polyethylene (PE) powder with bran without adding any proadhesive. Due to the risk of thermal decomposition of lignocellulose components, resulting in intense gas release, low temperatures were used during the process.

### 2.2. Materials

The test samples were made according to a low-density polyethylene injection method with a wheat bran filler from a local mill near the city of Lublin (Poland). The polyethylene powder Dowlex 2631.10UE [77], manufactured by The DOW Chemical Company (Schkopau, Germany), was used. This material is intended for the injection of elements which require high dimensional accuracy and for the rotational casting of thin-walled elements.

The polyethylene was filled with wheat bran obtained from a local mill i.e., wheat grain shells, which are separated as waste when the grains are ground into white wheat flour. They consist mainly of crude fiber, which includes fibrous substances such as cellulose, lignin and hemicellulose. Other ingredients of wheat bran are phytic acid, oligosaccharides, nonstarch polysaccharides as well as fats and proteins [70,78].

### 2.3. Research Programme and Methodology

Before use, the bran was ground into a fine powder in a grinding mill, and then subjected to a drying procedure in a laboratory drier for 24 h at 50 °C. Then, using a shaker equipped with a column of sieves with a mesh size of 0.8, 0.6 and 0.4 mm, three fractions with grain sizes < 0.4 mm, 0.4–0.6 mm and 0.6–0.8 mm were separated.

The experimental studies were carried out according to the complete plan, in which the following variable factors were adopted:

(1) Content by weight of bran: 0%, 5%, 10%, 15%;
(2) The grain size of the bran fraction used <0.4mm, 0.4–0.6 mm and 0.6–0.8 mm.

In order to produce the measurement samples, polyethylene powder and individual fractions of ground wheat bran in the amount of 5%, 10% and 15% by weight were mechanically mixed in a planetary mixer and then injected under the conditions given in Table 1. As a result, nine measurement series of biocomposite samples were obtained and one control series of unfilled polyethylene.

Some of the samples were tested 24 h after their production, while the rest only after the natural aging process. The samples were subjected to natural aging as a result of storage for 2.5 years in a dark room at a temperature of 20–25 °C and a humidity of about 55%, where they were not exposed to UV radiation or any chemicals.

**Figure 1:**
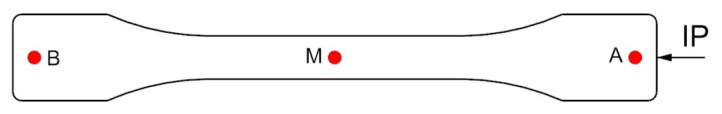
Scheme of sample measurement with marked points where microscopic observations as well as color and gloss measurements were carried out: IP—injection point; A—gate side; M—middle; B—opposite side of the gate.

The conducted experimental studies included:

- Observation of the sample surface with the optical microscope (Nikon, Tokyo, Japan), model Eclipse LV100ND equipped with a DS-U3 camera using the NIS-Elements AR 4.20.00 software (Nikon, Tokyo, Japan). The transmitted light method was used for observation. Observations were made at three points of the sample, marked in Figure 1. All samples were observed in identical magnification and illumination parameters.

- Measurement of the color of samples in accordance with ASTM E308 [79], for which the Ci4200 spectrophotometer (X-Rite, Grand Rapids, MI, USA) was used. The color is described in the CIELab system, where it is defined in the L*, a*, b* area. Parameter a* describes the color from green (negative values) to red (positive values); parameter b*, the color from blue (negative values) to yellow (positive values); and parameter L* is luminance–brightness, representing the gray scale from black to white (0 is black and 100 is white). The difference between the two colors—two points in the three-dimensional space L*, a*, b* are described by the relationship:
  $$\Delta E=\sqrt{\Delta L^{2}+\Delta a^{2}+\Delta b^{2}}$$
  where: ΔL, Δa and Δb, respectively, represent the difference in color parameters between the compared samples. The measurements of the color of the injection mouldings were taken at points A and B and marked in Figure 1;

- Measurement of the gloss of the surface of samples using the X - Rite Ci4200 spectrophotometer, according to ISO 2813: 2001 [80] at an angle of 60° of the aperture of the image of the light source and the receiver. Gloss of injection moulded parts was measured at points A and B, as marked in Figure 1;
- Measurement of longitudinal processing shrinkage of samples according to ISO 294-4: 2005 [81];
- Determination of tensile strength σ (MPa), nominal elongation at maximum tensile stress ε (%) and Young’s modulus (MPa) using Z010 testing machine (Zwick/Roell, Ulm, Germany) according to ISO 527-2 [82] at a tensile speed of 50 mm/min;
- Determination of the mass melt flow rate MFR (150 °C/2.16 kg) in g/10 min. The measurement was carried out using a load plastometer MeltFlow TQ6841 model (CEAST, Turin, Italy) based on the recommendations of ISO 1133-1: 2011 using method A [83];
- Determination of the relationship between pressure p specific volume v and temperature T during isobaric cooling in accordance with ISO 17744: 2004 [84]. The measurements were carried out with the p-v-T 100 device from SWO Polymertechnik GmbH (Krefeld, Germany). The test samples were heated to the maximum measurement temperature of 155 °C and compressed to the preset pressure value (20, 40, 60, 80, 100 and 120 MPa). Then, the set pressure was kept constant and the samples were cooled at a rate of 5 °C/min to a temperature of 35 °C while the changes in specific volume were measured, and the process was repeated for the next higher compression pressure value.
- FTIR analysis of samples using a FTIR TENSOR 27 spectrophotometer (Bruker, Germany) equipped with an attenuated total reflection (ATR) attachment with diamond crystal. Spectra were collected in the range of 600–4000 cm^−1^ with 16 scans per sample with a resolution of 4 cm^−1^.
- Differential scanning calorimetry (DSC) tests of the obtained injection mouldings using the DSC 204 F1 Phoenix differential scanning calorimeter (NETZSCH, Günzbung, Germany) and the NETZSCH Proteus data processing software (NETZSCH, Günzbung, Germany), according to ISO 11357-1: 2016 [85]. The DSC curves were recorded in the heating (I) cycle from −150 to 150 °C (at a rate of 10 K/min), cooling from 150 to −150 °C (at a rate of 10 K/min) and heating (II) −150 to 150 °C (at a rate of 10 K/min). The samples were tested in aluminium crucibles with a pierced lid. On the basis of the DSC curves, the degree of crystallinity *X_c_*, the enthalpy of melting *ΔH_m_*, the melting point *T_m_*, the crystallization temperature and the glass transition temperature *T_g_* of the injection mouldings were determined. The degree of crystallinity was calculated from the relationship
  $$X_{c}=\left(\frac{\Delta H}{\left(1-u\right)\times \Delta H_{100\%}}\right)\times 100\%$$
  assuming that for PE ΔH_100%_ = 293 J/g [86]. The inflection point of the DSC curve in the area of the glass transition was taken as the glass transition temperature.

- Thermogravimetric analysis (TG), carried out using the STA 449 F1 Jupiter thermal analyzer (NETZSCH, Günzbung, Germany). The tests were carried out at temperatures of 30–700 °C in an atmosphere of synthetic air with a gas flow rate of 20 mL/min and the heating rate 10 °C/min. The samples weighing about 10 mg were tested in crucibles made of Al_2_O_3_.

## 3. Results

The test results were statistically analyzed in the STATISTICA 13 program (StatSoft, Tulsa, OK, USA). In order to determine whether there were significant differences between the compared results, the ANOVA analysis of variance was used. Before that, the required assumptions, such as the normality of the distribution of variables, were checked (W. Shapiro–Wilk test), and the homogeneity of variance (Levene’s or Brown Forsythe’s tests). The Welch test was used in the few cases where heterogeneity of variance was found. However, when the variables did not meet the condition of normal distribution, the nonparametric Kruskal–Wallis test was used. When the above-mentioned analyses confirmed the presence of statistically significant differences, the Tukey’s multiple comparison test was performed. This test, called the posthoc test, enables the grouping of means and isolating of homogeneous groups. The significance level *p* = 0.05 was adopted in the analyses. The obtained results are presented in the form of graphs, in which the mean values and their standard deviations were presented.

### 3.1. Visual Characteristics of Injection Mouldings 

**Figure 2:**
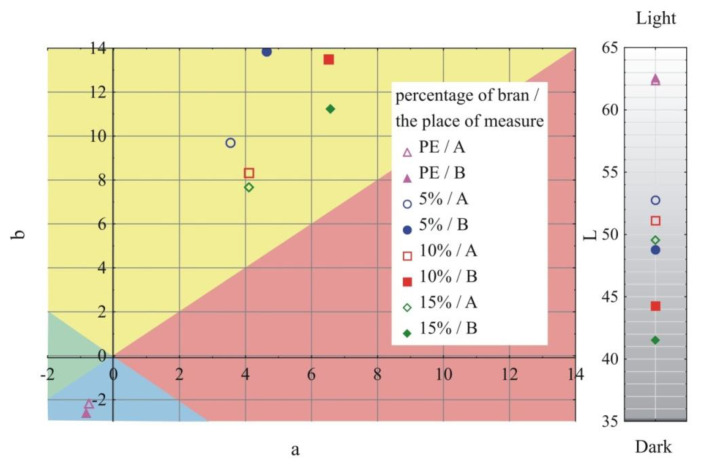
Variability of the color parameters L, a, b of injection mouldings depending on the mass fraction of bran and grain size < 0.4 mm at measuring points A and B (position of measuring points marked in Figure 1).

**Figure 3:**
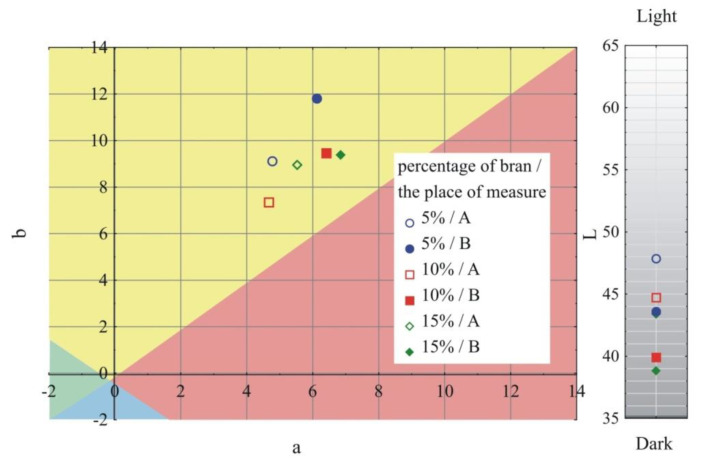
Variability of the color parameters L, a, b of injection mouldings depending on the content of the mass fraction of bran and grain size 0.4–0.6 mm at measuring points A and B (position of measuring points marked in Figure 1).

**Figure 4:**
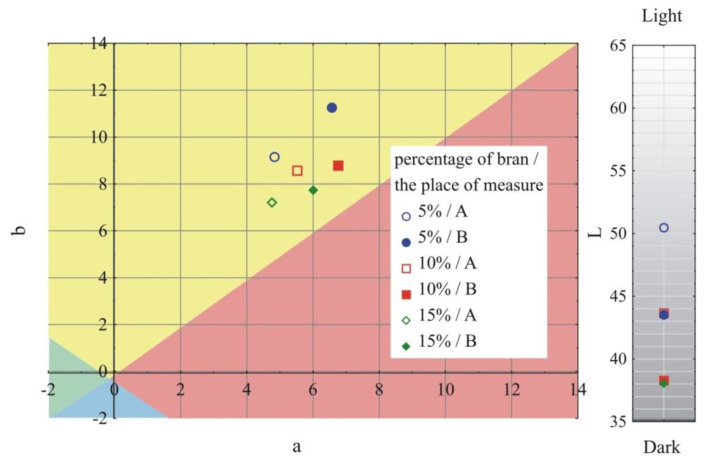
Variability of the color parameters L, a, b of injection mouldings depending on the content of the mass fraction of bran and grain size of 0.6–0.8 mm at measuring points A and B (location of measuring points marked in Figure 1).

**Figure 5:**
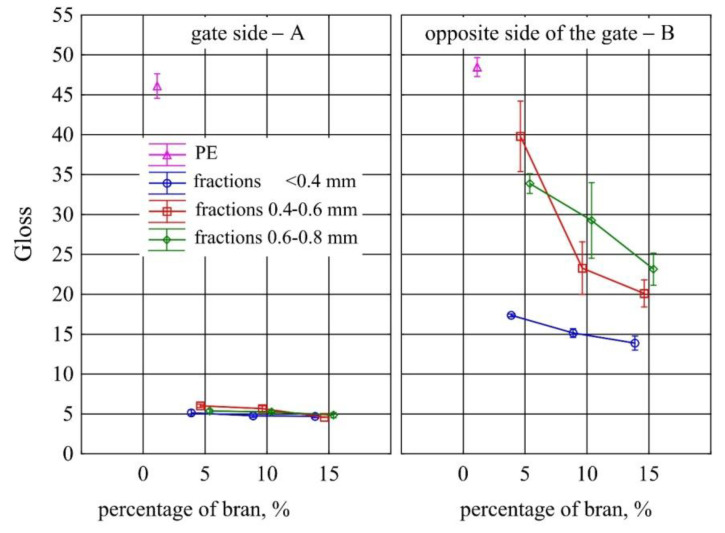
Variability of the gloss of injection mouldings depending on the mass content and grain size of the bran fraction used, on the side of the material supply through the gate—A and on the side opposite to the gate—B.

The results of the color and gloss measurements are shown in Figure 2, Figure 3, Figure 4 and Figure 5. Color parameters L, a, b of the injection mouldings are shown in Figure 2, Figure 3 and Figure 4, grouped into the mass content and the grain size of the introduced bran fraction. For comparative purposes, Figure 2 also shows the results obtained for mouldings made of polyethylene alone. Measurements were carried out in two parts of the mouldings marked at the measurement points (Figure 1), A—on the expansion of the mouldings on the side of the plastic supply through the pinpoint gate and B—on the expansion of the mouldings on the side opposite to the pinpoint gate, at the end of the moulding cavity. It was observed that introducing 5% bran content into polyethylene caused a distinct color change ΔE = 15.9–19.2. The color of the samples changed towards the shades of yellow and red, and at the same time, the samples darken, as evidenced by the decrease in the luminance L value. Differences in the color of the compacts related to the grain size of the introduced bran fraction were also observed. With the increase in the size of the grains while maintaining the same mass content, the differences in color increased, starting from the means 2 < ΔE < 3.5, recognizable by the inexperienced observer, through the clear differences 3.5 < ΔE < 5, and in some cases even large differences ΔE > 5. They are mainly caused by the darkening of the samples, which is reflected in the decrease in luminance L. Within the individual bran fractions, increasing their mass content from 5% to 10% caused a color change towards blue shades, and the b parameter decreased. These changes can be classified from average ΔE = 2.2 for the smallest bran fraction < 0.4 mm and ΔE = 3.6 for the 0.4–0.6 mm fraction, to large color changes ΔE = 6.9 at the largest fraction 0.6–0.8 mm. A further increase in the content of bran from 10% to 15% had a much smaller effect on the color change of the samples. In most cases, they fell within the range 1 < ΔE < 2, which is recognizable only by an experienced observer.

**Figure 6:**
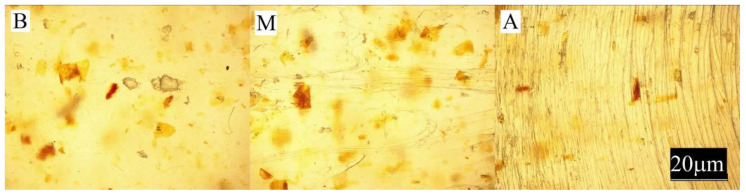
Appearance of the individual parts of the injection moulding obtained with a 5% content of bran fraction with grain size < 0.4 mm at measuring points A, M, B (the positions of the points are marked in Figure 1).

**Figure 7:**
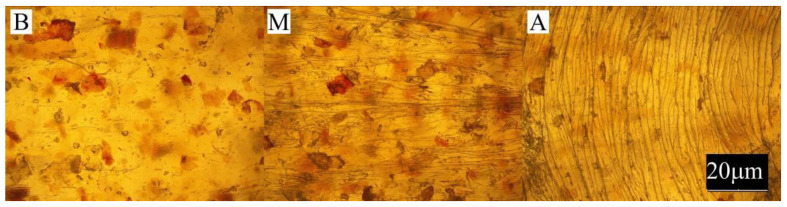
Appearance of the individual parts of the injection moulding obtained with a 15% content of bran fraction with grain size < 0.4 mm at measuring points A, M, B (the positions of the points are marked in Figure 1).

**Figure 8:**
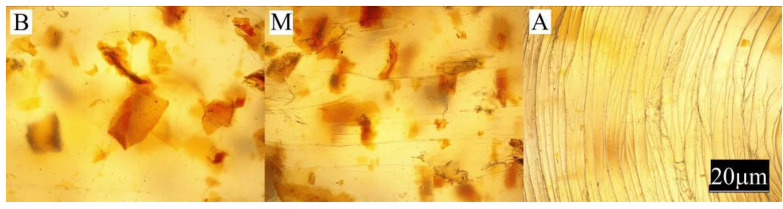
The appearance of individual parts of the injection moulding obtained with a 5% content of bran fraction with a grain size of 0.4–0.6 mm at measuring points A, M, B (the position of the points is marked in Figure 1).

**Figure 9:**
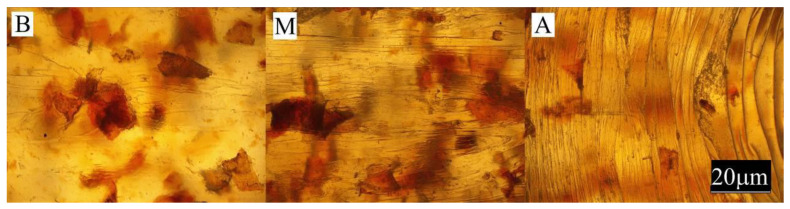
The appearance of individual parts of the injection moulding obtained with a 15% content of bran fraction with a grain size of 0.4–0.6 mm at measuring points A, M, B (the position of the points is marked in Figure 1).

**Figure 10:**
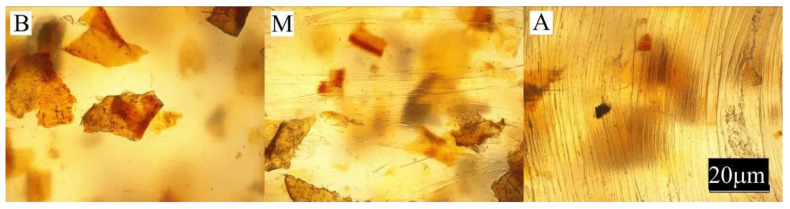
The appearance of individual parts of the injection moulding obtained with a 5% content of the bran fraction with a grain size of 0.6–0.8 mm at measuring points A, M, B (the positions of the points are marked in Figure 1).

**Figure 11:**
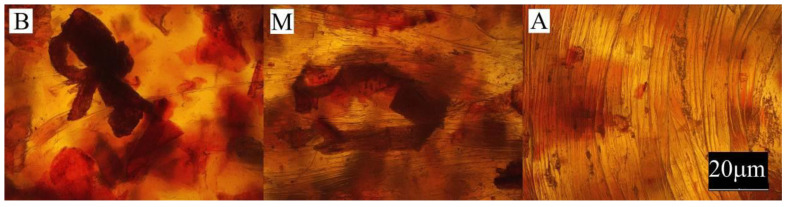
The appearance of individual parts of the injection moulding obtained with a 15% content of the bran fraction with a grain size of 0.6–0.8 mm at measuring points A, M, B (the positions of the points are marked in Figure 1).

Large changes in color were found along the length of the mouldings—along the flow path of the material (Figure 2, Figure 3 and Figure 4), which was manifested by different values of the color parameters determined for points A and B. The color of the mouldings became darker on the flow path, as evidenced by the reduction in the luminance L, and changes towards shades of yellow, and parameter b increased. The described differences were most pronounced in the case of the smallest fraction of bran with grain size < 0.4 mm, where ΔE = 6 with 5% content and, respectively, ΔE = 8.9 and ΔE = 9.1 with 10% and 15% of bran content. In the case of the fractions with larger grains, the observed color differentiation along the length of the mouldings was similar and amounted to approximately ΔE = 5.5—large color differences. Most likely, it is related to the different position of the filler grains along the flow path of the material. It is clearly seen in Figure 6, Figure 7, Figure 8, Figure 9, Figure 10 and Figure 11 that with a growing distance from the injection point, more and more grains were located at the surface of the sample. Due to their structure, the bran can scatter light rays, which is perceived as a decrease in the brightness at point B of the injection mouldings. At this point, it should be noted that the influence of wheat bran on the color of biocomposites based on polyethylene differs depending on their mass share. It is shown above that when filling with bran up to 15% by mass, the color darkened with an increase in the bran content, which may be associated with a gradual loss of transparency. On the other hand, with a much higher mass fraction of bran up to 50%, with the complete lack of transparency, increasing the mass fraction caused an increase in the brightness of the color of biocomposites based on polyethylene [70].

**Figure 12:**
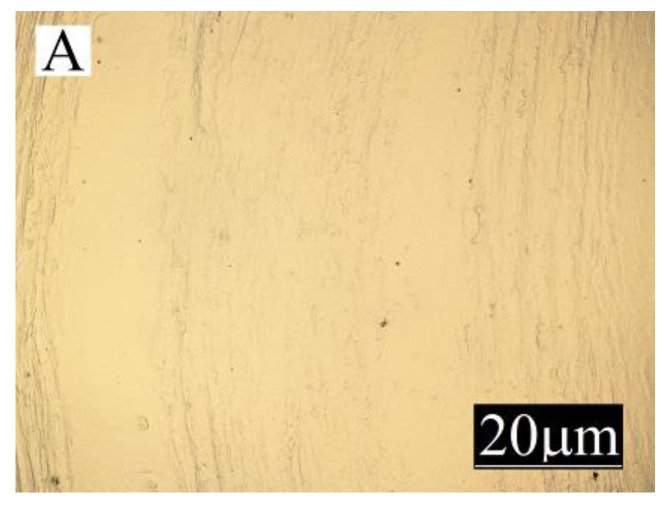
Appearance of the unfilled polyethylene moulding at measuring point A (the position of the point is marked in Figure 1).

Large changes in gloss were also observed along the length of the mouldings (Figure 5). The addition of bran caused a significant reduction in the gloss of the mouldings on the side of the plastic supply through the gate—point A. The gloss in this area from the value of 46 obtained for PE alone decreased to an average of five for all tested fractions and bran content. This is largely due to the presence of clear traces of the stream front of the material perpendicular to the direction of its flow (photo A in Figure 6, Figure 7, Figure 8, Figure 9, Figure 10 and Figure 11). These types of traces are most often caused by the pulsating flow of the material, which occurs especially when processing multiphase mixtures of plastics. In this case, the reason is the bran grains flowing through the pinpoint gate. For comparison, Figure 12 shows the appearance of the moulding in point A, made of unfilled polyethylene. In the central part of the mouldings (photo M in Figure 6, Figure 7, Figure 8, Figure 9, Figure 10 and Figure 11), these marks take the form of longitudinal lines. At point B of the part, the decrease in gloss value was much smaller. In this area (photo B in Figure 6, Figure 7, Figure 8, Figure 9, Figure 10 and Figure 11), the aforementioned traces were absent or barely visible. Posthoc analyses of the results of measurements carried out in this area of mouldings showed that the smallest comparable decrease in gloss was caused by a 5% addition of bran fractions with grain sizes of 0.4–0.6 mm and 0.6–0.8 mm. The largest decrease in gloss was observed for the fraction with the smallest grains < 0.4 mm in the range of 5%–15%. Statistically comparable values were obtained for the highest 15% content of the bran fractions with larger grains and 10% of the 0.4–0.6 mm fraction.

### 3.2. Mass Melt Flow Rate

**Figure 13:**
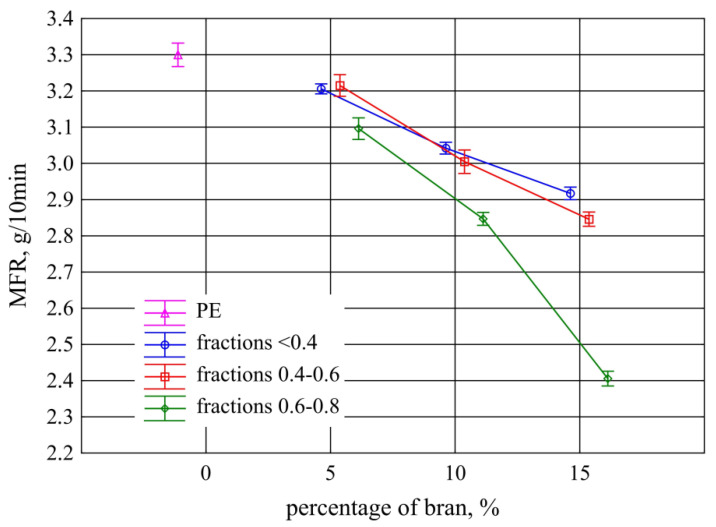
Mass flow rate of samples taken from injection mouldings depending on the mass content and grain size of the introduced bran fraction (average values with standard deviation).

The results of the research on the mass flow rate MFR of samples taken from injection mouldings are shown in Figure 13. Compared to polyethylene alone, for which MFR = 3.3 ± 0.1 g/10 min, a statistically significant decrease in the index value occurred from 10% in the case of fractions with grain size 0.4 mm and 0.4–0.6 mm, while for the 0.6–0.8 mm fraction from the content of 5%. The decrease in the value of the mass melt flow rate with increasing the filler content is the obvious effect of the increase in the viscosity of the composition due to the presence of fine particles dispersed in the polymer matrix [50,73,87,88,89]. A clear proportional decrease in the MFR value was observed, on average by 0.16 g/10 min with a 5% increase in the content of the two bran fractions 0.4 mm and 0.4–0.6 mm. Statistical analysis showed that the results obtained with both of these fractions are comparable. A greater decrease in the MFR value was observed for the fraction with a grain size of 0.6–0.8 mm, which is 0.35 g/10 min with an increase in the content by 5%. With the highest content of 15% of this fraction, the lowest MFR value was obtained and equalled 2.4 ± 0.07 g/10 min. Compared to the remaining fractions with smaller grains with the same content, it was a decrease by 0.48 g/10 min, while in relation to the lowest tested content of other fractions, it was a decrease by as much as 0.8 g/10 min (25%). This proves a significantly limited composition flow by large bran particles (0.6–0.8 mm) present in the fraction.

### 3.3. p-v-T Diagrams

**Figure 14:**
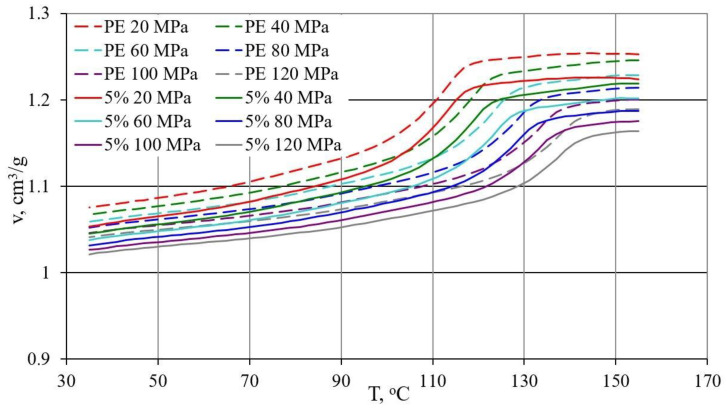
Dependence of specific volume v on temperature T for unfilled PE (dashed lines) and PE with 5% fraction content < 0.4 mm bran (continuous lines), at different pressure values.

**Figure 15:**
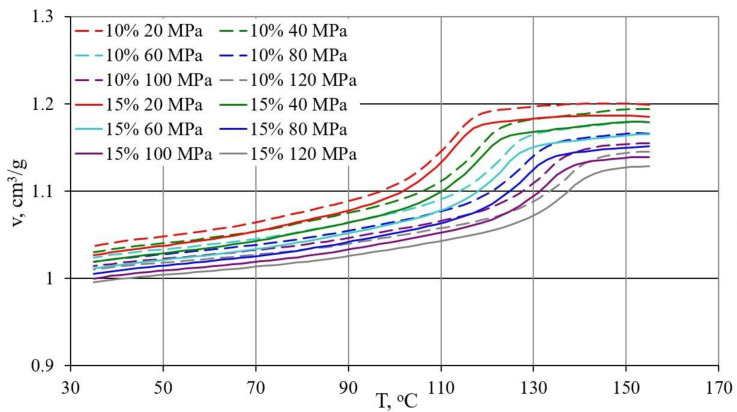
Dependence of specific volume v on temperature T for PE with 10% (dashed lines) and 15% (continuous lines) content of the fraction < 0.4 mm of bran, at different pressure values.

**Figure 16:**
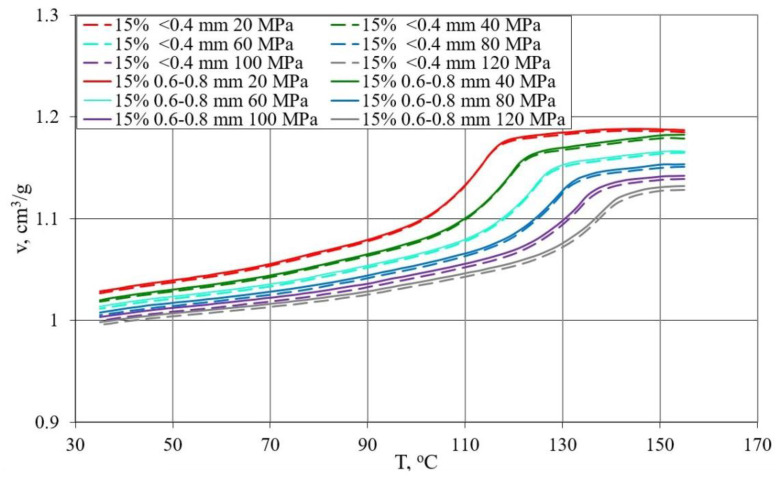
Dependence of specific volume v on temperature T for PE with 15% content of bran fraction < 0.4 mm (dashed lines), and 0.6–0.8 mm fraction (continuous lines) at different pressure values.

The results of the research on the relationship between pressure p specific volume ν and temperature T during isobaric cooling of the tested samples are shown in Figure 14, Figure 15 and Figure 16.

Figure 14 shows the results for PE alone and with 5% bran content in the form of p-ν-T graphs, while Figure 15 for polyethylene with 10% and 15% bran content.

The course of the curves obtained was similar, but it can be noticed that with increasing pressure, a distinct shift towards higher temperature values underwent a phase change—crystallization temperature, the curves of which corresponded to a rapid decrease in proper volume. The course of the curves differed more for higher pressure values. In the case of polyethylene alone, the reduction in the specific volume in the liquid state was from 0.002 cm^3^/g at the lowest pressure to 0.006 cm^3^/g at the highest pressure for each 10 °C during cooling. In the solid state, it was from 0.006 cm^3^/g at the highest pressure to 0.011 cm^3^/g at the lowest pressure, for each 10 °C. For polyethylene with the highest content of 15% bran, the reduction in the liquid specific volume was from 0.002 cm^3^/g at the lowest pressure to 0.0055 cm^3^/g at the highest pressure for each 10 °C reduction in temperature. However, in the solid state, it was from 0.006 cm^3^/g at the highest pressure to 0.01 cm^3^/g at the lowest pressure, for each 10 °C. Therefore, it can be concluded that the rate of changes in the specific volume along with the decrease in the temperature of PE itself and the examined content of bran is very similar. The specific volume of all tested samples in the solid state decreased with the temperature decrease slightly faster than in the liquid state.

Due to the content of bran, the decrease in specific volume in the area of phase transformation was clearly smaller, and due to the phase transformation taking place in a wider temperature range, it became decreasingly clear. These changes increase with increasing pressure. The reduction in specific volume as a result of cooling from 155 to 35 °C was at a pressure of 20 MPa for polyethylene 0.177 cm^3^/g (decrease by 16.5%), and for polyethylene with 15%, bran 0.159 cm^3^/g (decrease by 15.4%), while at a pressure of 120 MPa, 0.148 cm^3^/g (decrease by 14.2%) and 0.133 cm^3^/g (decrease by 13.3%).

The lower specific volume was that of polyethylene samples with the highest 15% bran content of 0.996 cm^3^/g (at a temperature of 35 °C and a pressure of 120 MPa). Under the same conditions, the specific volume of the samples with a content of 10% bran was 1.010 cm^3^/g, with a 5% hemline content of 1.021 cm^3^/g, and polyethylene alone, 1.040 cm^3^/g.

There was no effect of the applied bran fraction in the studied range on the determined p-v-T relationships. The obtained curves (Figure 16) with the highest 15% content of both fractions with grain sizes < 0.4 mm and 0.6–0.8 mm had almost the same course, and small differences were included in the measurement errors.

### 3.4. Chemical Structure

**Figure 17:**
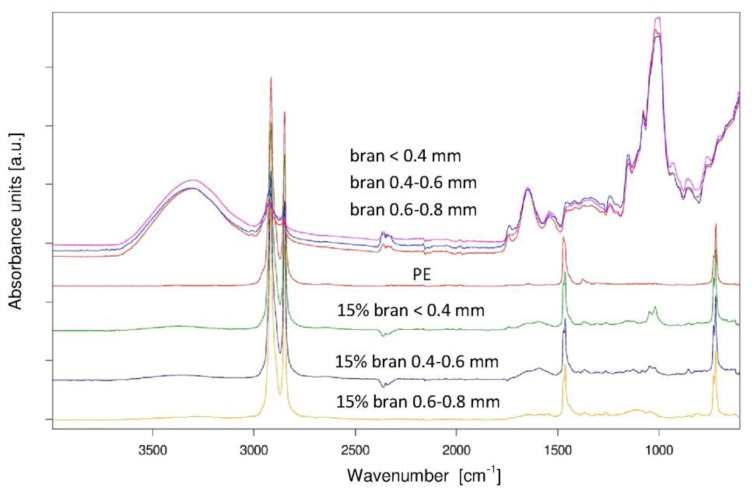
ATR-FTIR spectra of bran with different granulation, PE and PE pomace containing 15% by weight of bran.

In order to verify the impact of the injection process and the addition of a different amount of biofiller with different granulation on the chemical structure of polyethylene extrudate, an ATR-FTIR analysis was performed. FTIR spectra made for brans (Figure 17) with different granulation were identical. The presence of characteristic absorption bands on the spectra confirms that, regardless of their granulation, bran consists of polysaccharides, phenolic and lipid compounds and proteins, as well as absorbed water [78]. The −OH groups present in polysaccharides, polyphenols and water gave an absorption spectrum at 3311 cm^−1^. Vibrations of −OH groups from water absorbed by starch appeared on the spectrum at 1648 cm^−1^ [90,91]; however, these broad absorption spectra may also come from vibrations of carboxylate groups (present in the pectin structure), vibrations of phenyl rings (present in polyphenols and lignin) and the amide C-N group present in proteins [91]. The absorption spectra at 1546 cm^−1^ resulting from the vibrations of the N-H amide group also indicate the presence of protein in the bran composition [92]. The absorption spectra at 1743 cm^−1^ came from the vibration of the C=O groups present in the carbonyl compounds building the structure of pectins and hemicellulose [91,93]. The presence of starch, cellulose, hemicellulose, pectin and lignin in the bran is confirmed by the absorption spectra visible at 1150, 1078, 1017 and 999 cm^−1^, which came from the vibrations of the C−O−C and C−O groups.

On the other hand, the FTIR spectrum of polyethylene (Figure 17) showed characteristic absorption spectra at 2916, 2849, 1471 and 1377 cm^−1^, resulting from stretching and deformation vibrations of methylene groups. Additionally, the presence of the absorption spectra at 1377 cm^−1^ proves the presence of branches in the structure of the linear PE. In the FTIR spectra of PE extrudate with bran (Figure 17), absorption bands are observed in the spectra of both components. However, the absorption bands were due to the vibrations of the C−O−C and C−O groups and were shifted slightly towards higher wavenumbers.

### 3.5. Thermal Properties

**Figure 18:**
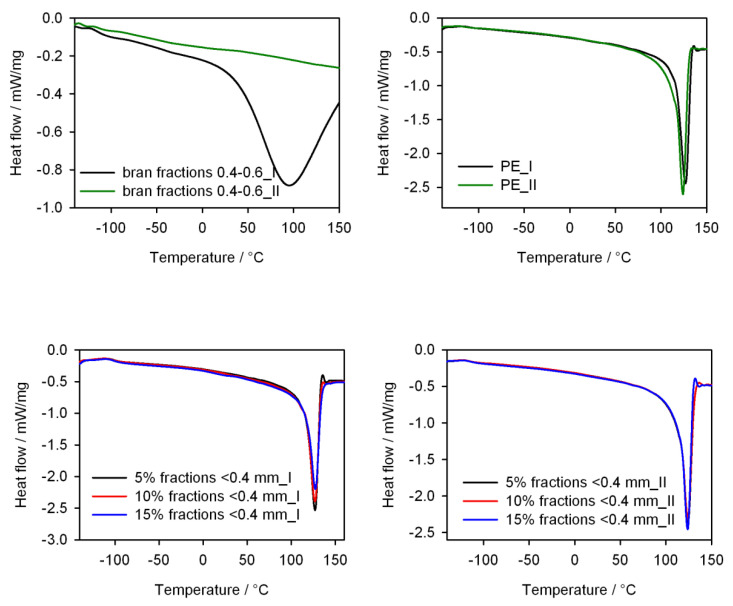

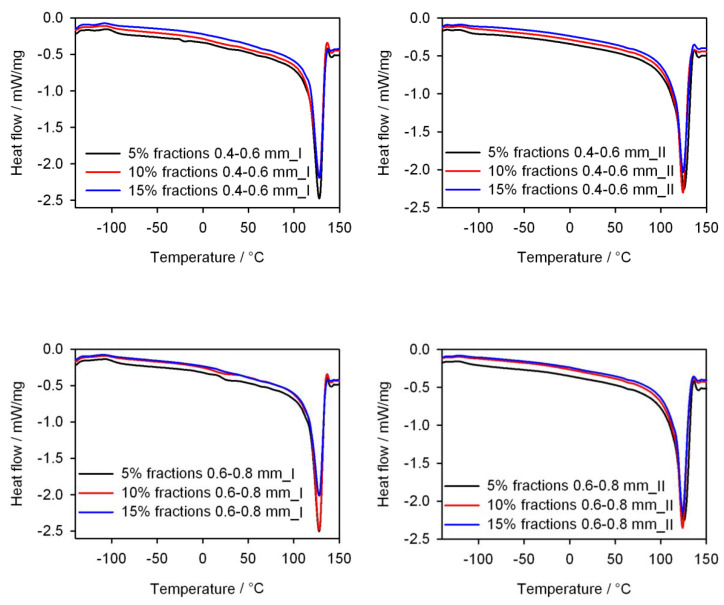
DSC curves from the first and second heating cycle of components and composites with different bran content.

In order to determine the effect of the amount of bran of various sizes on the glass transition temperature, crystallization temperature, melting point and crystallinity of injection mouldings, tests were carried out using differential scanning calorimetry. Table 2 presents numerical data characterizing selected thermal properties and the level of crystallinity, calculated on the basis of DSC curves (Figure 18). The glass transition temperatures of the samples determined from the first heating cycle were noticeably higher than for pure PE. However, the T_g_ values determined for the samples from the second heating cycle, after removing their thermal history, were almost identical for PE and for samples filled with bran with the smallest granulation. Higher T_g_ values were maintained for samples obtained from bran with higher granulation.

Compared to pure polyethylene, the melting point of the composites, determined from both the first and second heating cycles, was slightly higher, whereas the crystallization temperature was lower. More noticeable differences in T_m_ values were visible in the case of the first heating cycle. This increase may indicate that the structure of the mouldings contains larger PE crystallites than in pure PE. On the other hand, after cooling the samples and reheating, the internal structure was probably rearranged and smaller PE crystallites formed, and thus the T_m_ of the extrudate is comparable to the T_m_ of pure PE. On the other hand, the observed degree of crystallinity was clearly higher than for pure PE, and the X_c_ values determined from the second heating cycle were greater than those from the first heating cycle. In a previous work [70], the effect of bran addition on X_c_ of PE expeller was presented, and we noticed that the X_c_ values were lower for the extrudate than for the initial PE. However, it should be taken into account that the amount of biofiller used was much higher. Previously, it was as much as 50% of bran, whereas in this case, 5%–15% by weight was used. The hydrophilic structure of the bran, mainly due to the presence of hydroxyl groups in their chemical structure, is not fully compatible with the structure of PE chains, which probably hinders the formation of PE crystallites on the bran surface [94]. However, when using up to 20% of bran, they act as a kind of separator. They separate low-density polyethylene chains that have a significant amount of short-chain branching hindering crystallization, allowing the formation of crystallites from long-chain PE fragments.

In the DSC curves (Figure 18), there was one more transition region in the temperature range 30–50 °C from the first heating cycle. The most visible phase transition in this temperature range was shown by the extrudates containing the bran of the highest granulation. The lack of this transition in the DSC curves from the second heating cycle may suggest that it is related to the local melting of the smallest lamella areas, as postulated by the authors of the study [95]. A similar effect was also observed for PE composites with halloysite nanotubes [96].

**Figure 19:**
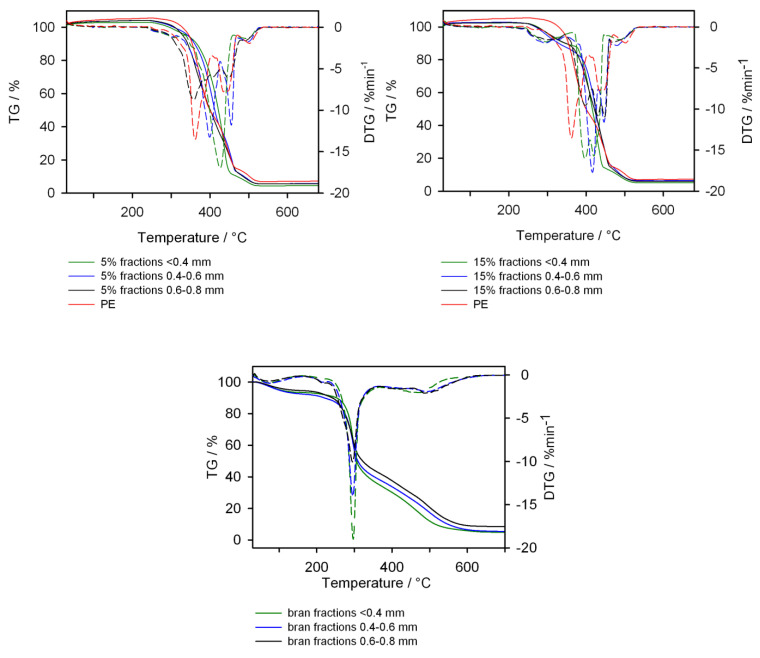
TG (continuous line) and DTG (dashed line) curves of components and composites with different bran content.

The thermal stability of the obtained biocomposites was determined on the basis of a thermogravimetric analysis carried out in an atmosphere of synthetic air. Figure 19 shows the TG and DTG curves of bran with different granulation and PE and the samples obtained with 5% and 15% by mass of filler. On the basis of the TG curves, it can be concluded that the thermal resistance of the bran is not influenced by its particle size. For all bran fractions, a 6% weight loss to the temperature of about 150 °C was observed on the TG curves. This is related to water evaporation [70]. At the temperature of about 170 °C, the TG curves of bran showed a loss in mass related to the processes of their degradation. Under measurement conditions, polyethylene presented thermal stability up to 270 °C; therefore, when selecting the processing temperature, the thermal resistance of the biofiller was of prime importance. Considering the TG curves of biocomposites containing bran with different particle sizes, it can be concluded that both samples containing 5% by weight and 15% by weight of bran are thermally stable up to about 250 °C. The first weight loss, whose maximum rate was observed at about 290 °C, was related to the degradation of the biofiller. It was 5% for samples containing 5% bran and 16% for samples containing 15% bran. Such a loss of mass was not observed on the TG curve of pure PE, which was thermally more resistant than the tested composites. However, the course of TG curves of pure PE and its composites is surprising. The rate of thermal decomposition of PE was higher than for composites. An expression of this difference may be the temperature at which 50% of the sample decomposed (T50%). For PE, T50% was 406 °C, for composites containing 15% bran with increasing particle size: 416, 421 and 424 °C, and for composites containing 5 bran: 421, 412 and 400 °C, respectively. Only the extrudate containing 5% bran with the largest grain diameter had a lower T50% value than PE. The remaining extrudates decomposed slower than PE, which was already explained by the slower diffusion of heat into the interior of the sample, especially at higher temperatures, and on the other hand, by hindered diffusion from the interior of the sample by products of gaseous decomposition [70].

### 3.6. Longitudinal Contraction

**Figure 20:**
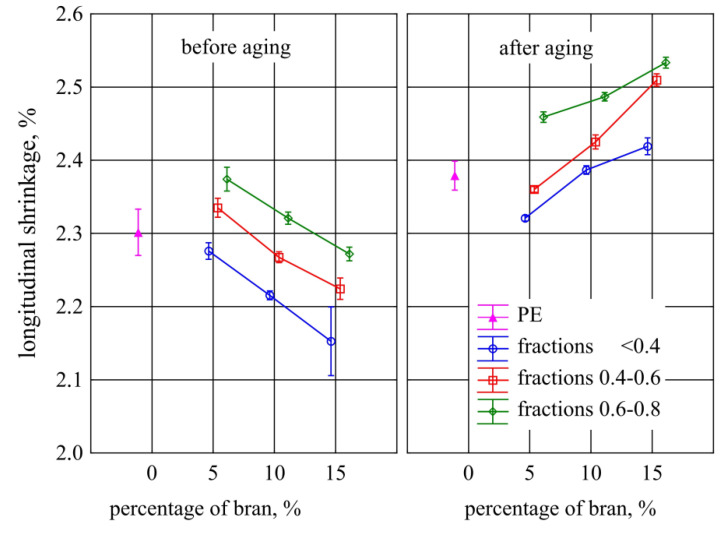
Longitudinal shrinkage of mouldings before and after aging depending on the content and grain size of the introduced bran fraction (average values with standard deviation).

The influence of the mass content and grain size of the introduced bran fraction on the longitudinal shrinkage of the mouldings is shown in Figure 20. The nonparametric statistical tests performed showed that there were significant differences between the shrinkage values of the compared samples. Subsequent posthoc tests showed that the longitudinal shrinkage of samples made of polyethylene alone before aging was not significantly different from that occurring after aging, and that the introduction of bran within the tested range did not significantly affect the shrinkage of the samples before aging. However, there were significant differences in shrinkage between samples before aging with the content of 5%–15% of the smallest bran fraction <0.4 mm and samples after aging with the highest content of 15% of the fraction 0.4–0.6 mm and the fraction 0.6–0.8 mm in the entire range of 5%–15%. The shrinkage of samples before aging containing 15% bran of all types of fractions also differed significantly from that occurring after aging in samples containing 5%–15% of the largest fraction 0.6–0.8 mm and the highest 15% fraction content of 0.4–0.6 mm. The lowest shrinkage value of 2.15 ± 0.05% was observed in samples before aging with 15% bran content of the smallest fraction < 0.4 mm, while the highest value of 2.53% ± 0.01% in the samples after aging with 15% content of the largest fraction of bran, 0.6–0.8 mm.

If the filler used is not subject to the processing shrinkage to the same extent as the polymer matrix, it leads to a shrinkage of the material on the filler grains by compressive stress, while the matrix by tensile stresses. Long-term exposure to compressive stresses could lead to the collapse of the bran particles, which at least partially explains the obtained results of the shrinkage of the mouldings [97].

### 3.7. Static Tensile Test

**Figure 21:**
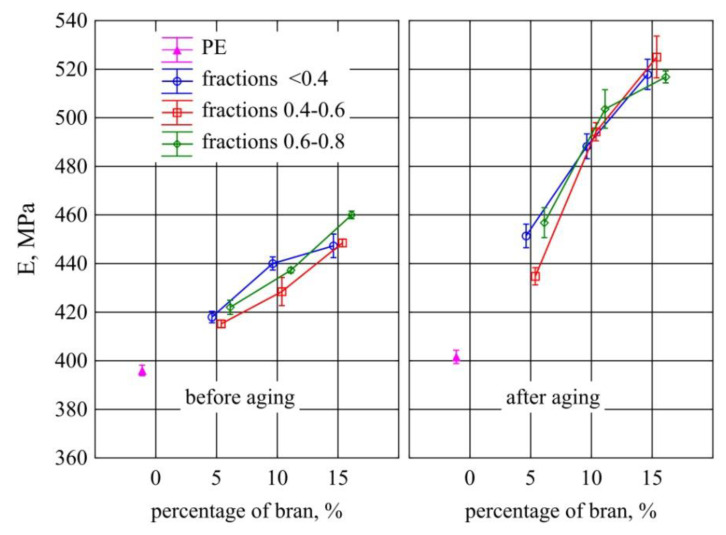
Young’s modulus of injection mouldings before and after aging depending on the mass content and grain size of the introduced bran fraction (average values with standard deviation).

The obtained results of the Young’s modulus measurements for the tested samples are shown in Figure 21. The tests showed that a significant increase in the modulus of the tested samples, compared to those made of PE alone, occurred after introducing at least 10% of bran. Before aging, a significant increase in the modulus for all wheat bran particles sizes by an average of 34 MPa occurred between the extreme contents of 5% and 15% of bran. Comparing the obtained results after the aging, it was found that from 10% of the bran content, there was a significant increase in the Young’s modulus in comparison to the samples before the aging. Aging caused a marked increase in the modulus along with the content of all examined bran fractions. The maximum difference between the 5% and 15% samples was 71 MPa on average. On the other hand, no effect of the size of the bran grains on Young’s modulus before and after aging was observed. The values of Young’s modulus of samples from polyethylene alone before and after aging also showed no significant differences.

**Figure 22:**
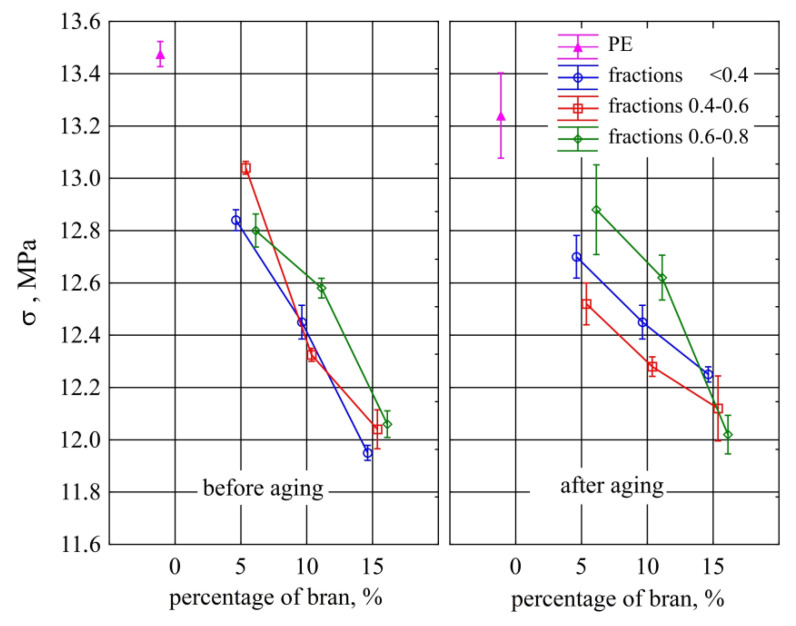
Tensile strength of injection mouldings before and after aging depending on the mass content and grain size of the introduced bran fraction (average values with standard deviation).

The conducted tests showed a statistically significant reduction in tensile strength of the samples with the highest content of 15% with all the tested bran fractions compared to those made of polyethylene alone (Figure 22), both before and after aging. This reduction was on average 1.46 MPa for samples before aging and 1.11 MPa for samples after aging. However, the posthoc tests did not show any significant differences in the strength of the samples made of polyethylene alone before and after aging, or the aging effect on the strength of the compared samples with the same content and size of bran grains. On the other hand, there were differences between the samples before aging with the lowest 5% and the highest 15% content of all examined bran fractions. These samples, after aging, showed no significant differences in strength. However, an increase in the dispersion of the obtained measurement results was observed in their case.

**Figure 23:**
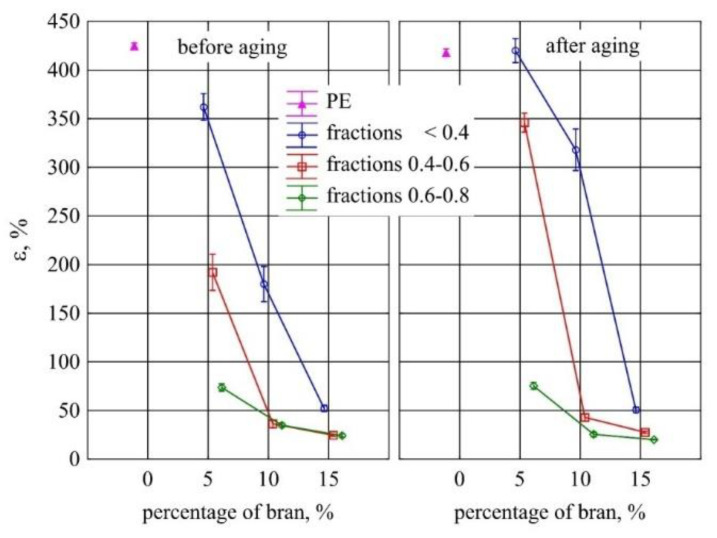
Elongation at break of injection mouldings before and after aging depending on the mass content and grain size of the introduced bran fraction (average values with standard deviation).

A similar effect of the tested factors was observed on the elongation at break of the tested specimens (Figure 23). The elongation values of polyethylene mouldings alone before and after aging did not differ. A statistically significant reduction in the elongation at break of the samples as compared to PE alone was observed after the introduction of 10% and 15% bran from all tested fractions. As the content of the bran fraction with the smallest particles <0.4 mm increased, the elongation at break decreased to the greatest extent from 360% to 52%. For the 0.4–0.6 mm fraction, the maximum reduction in elongation before aging was 168%, while for the 0.6–0.8 mm fraction, it was only 49%. In most cases, there was no statistically significant effect of aging on the elongation at break of the compared samples with the same content and size of the bran fraction.

Lignocellulose is characterized by better stiffness than most of the polymeric materials commonly used for the production of biocomposites with natural fillers [98,99]. Therefore, increasing the filler content increases the value of the modulus of elasticity. In the absence of modification of the adhesion between the polymer matrix and the filler, despite the increase in stiffness, the brittleness increases, which is manifested in a reduction in tensile strength and often a significant reduction in deformability. The nature of the fracture itself also changes from plastic to brittle [32,46,55,67,99,100]. Significant differences in the values of Young’s modulus before and after aging may be related to the observed changes in the longitudinal contraction of mouldings in the analyzed period of time. The potential collapse of the filler grains during the aging period could lead to an increase in internal stresses [90], which translates to the observed increase in stiffness.

## 4. Conclusions

The conducted research showed a significant influence of wheat bran on the appearance of the obtained injection mouldings. There were both clear changes in the color towards shades of yellow and red with an increase in the content of bran, and significant differences in the color and gloss of injection mouldings along with the distance from the injection point. In the immediate vicinity of the gate, clear traces of pulsating flow of the stream of the material were observed, affecting the visual quality, which blurred with distance, affecting the gloss gradient along the length of the samples. The intensity of the observed pulsating flow lines depended on both the content and the size of the filler grains. Moreover, the farther away from the gate, the more filler grains accumulate at the surface of the forming cavity, thus affecting the color perception. The obtained results of the research on visual properties suggest that when designing moulds intended for injection of biocomposites with a natural filler, it will be most advantageous to locate point gates at the bottom of the manufactured elements. The lines formed during the pulsating flow of the material will not adversely affect the visual perception of the finished products. Due to this, the dull and rougher area around the gate will be located in a less visible place, and the flow lines will be blurred before reaching the places visible during standard use. However, the color in the most visible places will be more uniform. The melt flow rate, which is the primary index of processability, decreased with increasing bran content, due to an obvious increase in the viscosity of the composition. Nevertheless, the composition can be easily processed by injection moulding, and the obtained MFR values do not constitute a prerequisite for the use of processing aids with the analyzed filler contents.

The p-v-T tests showed that the content of bran reduces the decrease in specific volume during crystallization, which translates into lower processing shrinkage during injection. Measurements of processing shrinkage after a long storage period show that shrinkage progresses over time, which in turn may be the result of compressive stresses around the filler grains during the cooling of the compact.

The analysis of the chemical structure by means of FTIR spectroscopy shows the presence of components typical of lignocellulosic materials, but also the presence of water in the filler structure and branches in the polyethylene structure.

The obtained DSC results suggest the presence of changes in the polyethylene structure caused by the presence of bran, as evidenced by the differences in the melting point and the degree of crystallinity of the composites and unfilled PE during the 1st heating cycle, the absence of these differences during the 2nd heating cycle and the reorganization of the composite structure. Considering the thermal resistance of biocomposites under air atmosphere, it can be concluded that they are thermally stable up to about 250 °C. The first weight loss observed at about 290 °C on TG curves relates to the degradation of the biofiller; the second one at about 400 °C is connected with PE degradation.

The obtained values of tensile strength properties are typical for natural fillers, i.e., an increase in stiffness and brittleness as well as a decrease in tensile strength and deformability. The tests showed that for the tested contents, the deterioration of the tensile strength is on average about 1.4 MPa, i.e., 10% of the value for the content of 15wt.% of filler. The use of such a wheat bran content will allow the significant reduction in the production costs and polyethylene consumption while maintaining comparable strength.

It is important, however, that no deterioration of the mechanical characteristics of the tested composites after aging was observed. The tested materials are, therefore, suitable for use in mass production and do not lose their properties during long-term storage. As a result, the tested biocompositions may be suitable for the production of elements with seasonal use, which often require several months of storage.

## Figures and Tables

**Table 1:** Technological parameters of the injection process.

Parameter	Unit	Value
Injection pressure	MPa	100
Temperature of heating zones in plasticising system	°C	I—105 II—130 III—140
Injection nozzle temperature	°C	150
Mould temperature	°C	40
Injection cycle time	s	30
Cooling time in mould	s	20

**Table 2:** Glass transition, crystallization and melting temperatures and degree of crystallinity of polyethylene and its composites with bran obtained on the basis of DSC tests.

Sample	Heating I					Cooling	Heating II		
	T_g_ (°C)	T_m_ (°C)	ΔH_m_ (J/g)	X_c_ (%)	T_g_ (°C)	T_c_ (°C)	T_m_ (°C)	ΔH_m_ (J/g)	X_c_ (%)
PE	−109.1	126.5	161.6	55.2	−123.3	109.0	124.7	174.0	59.4
PE <04 5%	−101.2	127.0	164.7	59.2	−123.9	108.0	124.8	168.7	60.6
PE <04 10%	−102.1	128.2	157.4	59.7	−123.2	108.2	123.7	162.5	61.6
PE <04 15%	−101.8	126.7	150.4	60.4	−123.3	108.3	123.8	153.0	61.4
PE 04–06 5%	−97.8	128.3	161.1	57.9	−113.8	106.3	125.3	168.4	60.5
PE 04–06 10%	−100.5	127.8	150.8	57.2	−112.5	106.3	124.7	155.8	59.1
PE 04–06 15%	−100.9	128.6	146.8	59.0	−111.1	106.9	124.1	150.9	60.6
PE 06–08 5%	−99.0	128.3	161.6	58.1	−115.0	106.7	126.4	172.6	62.0
PE 06–08 10%	−97.8	126.9	164.6	62.4	−111.2	107.2	124.9	167.1	63.4
PE 06–08 15%	−101.0	128.3	144.7	58.1	−112.0	107.9	124.7	153.6	61.7

## References

[1] Melo P.M.A., Macêdo O.B., Barbosa G.P., Ueki M.M., Silva L.B. (2019). High-Density Polyethylene/Mollusk Shell-Waste Composites: Effect of Particle Size and Coupling Agent on Morphology, Mechanical and Thermal Properties. J. Mater. Res. Technol..

[2] Kumar N., Das D. (2017). Fibrous Biocomposites from Nettle (Girardinia Diversifolia) and Poly(Lactic Acid) Fibres for Automotive Dashboard Panel Application. Compos. Part B Eng..

[3] Xanthos M. (2010). Functional Fillers for Plastics.

[4] Sasimowski E., Majewski Ł. (2019). Effect of the Intensive Plasticizing Zone Design on the Effectiveness of Corotating Twin-Screw Extrusion. Adv. Polym. Technol..

[5] Liang J.Z. (2013). Reinforcement and Quantitive Description of Inorganic Particulate-Filled Polymer Composites. Compos. Part B Eng..

[6] Sasimowski E., Majewski Ł., Grochowicz M. (2019). Influence of the Conditions of Corotating Twin-Screw Extrusion for Talc-Filled Polypropylene on Selected Properties of the Extrudate. Polymers.

[7] Liu P. (2007). Polymer Modified Clay Minerals: A Review. Appl. Clay Sci..

[8] Väisänen T., Das O., Tomppo L. (2017). A Review on New Bio-Based Constituents for Natural Fiber-Polymer Composutes. J. Clean. Prod..

[9] Bher A., Unalan I.U., Auras R., Rubino M., Schvezov C.E. (2018). Roughening of Poly (Lactic Acid) and Thermoplastic Cassava Starch Reactive Blends Using Graphene Nanoplatelets. Polymers.

[10] Sánchez-Acosta D., Rodriguez-Uribe A., Álvarez-Chávez C., Mohanty A.K., Misra M., López-Cervantes J., Madera-Santana T.J. (2019). Physicochemical Characterization and Evaluation of Pecan Nutshell as Biofiller in A Matrix of Poly(Lactic Acid). J. Polym. Environ..

[11] Obasi H.C. (2015). Peanut Husk Filled Polyethylene Composites: Effect of Filler Content and Compatibilizer on Properties. J. Polym..

[12] Sikora J., Majewski Ł., Puszka A. (2020). Modern Biodegradable Plastics—Processing and Properties: Part I. Materials.

[13] Faruk O., Bledzki A.K., Fink H.P., Sain M. (2012). Biocomposites Reinforced with Natural Fibres: 2000-2010. Prog. Polym. Sci..

[14] Tian G., Zhuang J., Fu Y., Wang Z., Li Q. (2019). Enhanced Mechanical Strength of Polypropylene-Based Lignocellulosic-Plastic Composites by Cellulose Fibres. Bioresources.

[15] Bekele L.D., Zhang W., Liu Y., Duns G.J., Yu C., Jin X., Li X., Jia Q., Chen J. (2017). Preparation and Characterization of Lemongrass Fiber (Cymbopogon Species) for Reinforcing Application in Thermoplastic Composites. Bioresources.

[16] Baba B.O., Özmen U. (2015). Preparation and Mechanical Characterization of Chicken Feather/PLA Composites. Polym. Compos..

[17] Shaghaleh H., Xu X., Wang S. (2018). Current progress in production of Biopolymeric Materials Based on Cellulose, Cellulose Nanofibres, and Cellulose Derivatives. RSC Adv..

[18] Merkel K., Rydarowski H., Kazimierczak J., Bloda A. (2014). Processing and Characterization of Reinforced Polyethylene Composites Made with Lignocellulosic Fibres Isolated from Waste Plant Biomass Such As Hemp. Compos. Part B Eng..

[19] Das O., Bhattacharyya D., Hui D., Lau K.T. (2016). Mechanical and Flammability Characterisations of Biochar/Polypropylene Biocomposites. Compos. Part B Eng..

[20] Visakh P.M., Thomas S. (2010). Preparation of Bionanomaterials and Their Polymer Nanocomposites from Waste and Biomas. Waste Biomass Valorization.

[21] Ogunsona E.O., Codou A., Misra M., Mohanty A.K. (2019). A Critical Review on the Fabrication Processes and Performance of Polyamide Biocomposites from a Biofiller Perspective. Mater. Today Sustain..

[22] Yang H., Yan R., Chen H., Lee D.H., Zheng C. (2007). Characteristics of Hemicellulose, Cellulose and Lignin Pyrolisis. Fuel.

[23] Melo R.P., Marques M.F.V., Navard P., Duque N. (2017). Degradation Studies and Mechanical Properties of Treated Curauá Fibres and Microcrystalline Cellulose in Composites with Polyamide 6. J. Compos. Mater..

[24] Varshney V.K., Naithani S., Kalia S., Kaith B., Kaur I. (2011). Chemical functionalization of cellulose derived from nonconventional sources. Cellulose Fibres: Bio- and Nano-Polymer Composites.

[25] Mondal S. (2017). Preparation, Properties and Applications of Nanocellulosic Materials. Carbohydr. Polym..

[26] Wu C.S. (2015). Renewable Resource-Based Green Composites of Surface-Treated Spent Coffee Grounds and Polylactide: Characterization and Biodegradability. Polym. Degrad. Stab..

[27] Habibi Y., El-Zawawy W.K., Ibrahim M.M., Dufresne A. (2008). Processing and Characterization of Reinforced Polyethylene Composites Made with Lignocellulosic Fibres from Egyptian Agro-Industrial Residues. Compos. Sci. Technol..

[28] Tisserat B., Reifschneider L., Núñez J.C.L., Hughes S.R., Selling G., Finkenstadt V.L. (2014). Evaluation of the Mechanical and Thermal Properties of Coffee Tree Wood Flour—Polypropylene Composites. Bioresources.

[29] Andrzejewski J., Szostak M., Barczewski M., Łuczak P. (2019). Cork-Wood Hybryd Filler System for Polypropylene and Poly (Lactic Acid) Based Injection Molded Composites. Structure Evaluation and Mechanical Performance. Compos. Part B Eng..

[30] Formela K., Hejna A., Piszczyk Ł., Saeb M.R., Colom X. (2016). Processing and Structure-Property Relationship of Natural Rubber/Wheat Bran Biocomposite. Cellulose.

[31] Avérous L., Le Digabel F. (2006). Properties of Biocomposites Based on Lignocellulosic Fillers. Carbohydr. Polym..

[32] Montanes N., Garcia-Sanoguera D., Segui V.J., Fenollar O., Boronat T. (2018). Processing and Characterization of Environmentally Friendly Composites from Biobased Polyethylene and Natural Fillers from Thyme Herbs. J. Polym. Environ..

[33] Chafidz A., Rizal M., RM F., Kaavessina M., Hartanto D., Al Zahrani S.M. (2018). Processing and Properties of High Density Polyethylene/Date Palm Fiber Composites Prepared by A Laboratory Mixing Extruder. J. Mech. Eng. Sci..

[34] Jawaid M., Khalil H.P.S.A. (2011). Cellulosic/Synthetic Fiber Reinforced Polymer Hybrid Composites: A Review. Carbohydr. Polym..

[35] Sarsari N.A., Pourmousa S., Tajdini A. (2016). Physical and Mechanical Properties of Walnut Shell Flour-Filled Thermoplastic Starch Composites. Bioresources.

[36] Zhang Q., Li Y., Cai H., Lin X., Yi W., Zhang J. (2019). Properties Comparison of High Density Polyethylene Composites Filled with Three Kinds of Shell Fibres. Results Phys..

[37] Tufan M., Ayrilmis N. (2016). Potential Use of Halzenut Husk in Recycled High-Density Polyethylene Composites. Bioresources.

[38] Rahmat W., Sin T.L., Rahmat A.R., Isa N.M., Salleh M.S.N., Mokhtar M. (2010). Comparison of Rice Husk-Filled Polyethylene Composite and Natural Wood Under Weathering Effects. J. Compos. Mater..

[39] Majewski Ł., Gaspar Cunha A. (2018). Evaluation of Suitability of Wheat Bran as A Natural Filler in Polymer Processing. Bioresources.

[40] Šimkovic I., Kelnar I., Mendichi R., Bertok T., Filip J. (2017). Composite Films Prepared from Agricultural By-Products. Carbohydr. Polym..

[41] Matkó S., Toldy A., Keszei S., Anna P., Bertalan G., Marosi G. (2005). Flame Retardancy of Biodegradable Polymers and Biocomposites. Polym. Degrad. Stab..

[42] Gowman A.C., Picard M.C., Lim L.T., Misra M., Mohanty A.K. (2019). Fruit Waste Valorization for Biodegradable Biocomposites Applications: A Review. Bioresources.

[43] Yan L., Chouw N., Jayaraman K. (2014). Flax Fibre and Its Composites—A Review. Compos. Part B Eng..

[44] Lau K., Hung P., Zhu M.H., Hui D. (2018). Properties of Natural Fibre Composites for Structural Engineering Applications. Compos. Part B Eng..

[45] Saba J., Tahir P.M., Jawaid M. (2014). A Review on Potentiality of Nano Filler/Natural Fiber Filled Polymer Hybrid Composites. Polymers.

[46] Mirmehdi S.M., Zeinaly F., Dabbagh F. (2014). Date Palm Wood Flour As A Filler of Linear Low-Density Polyethylene. Compos. Part B Eng..

[47] Satyanarayana K.G., Monteiro S.N., Lopes F.P.D., Margem F.M., Santafe H.P.G., da Costa L.L., Kalia S., Kaith B., Kaur I. (2011). Dimensional analysis and surface morphology as selective criteria of lignocellulosic fibres as reinforcement in polymeric matrices. Cellulosic Fibres: Bio- and Nano-Polymer Composites.

[48] Murali Mohan Rao K., Mohana Rao K., Ratna Prasad A.V. (2010). Fabrication and Testing of Natural Fibre Composites: Vakka, Sisal, Bamboo and Banana. Mater. Des..

[49] Jagadish R.S., Raj B., Asha M.R. (2009). Blending of Low-Density Polyethylene with Vanillin for Improved Barrier and Aroma-Releasing Properties in Food Packaging. J. Appl. Polym. Sci..

[50] Santi C.R., Hage E., Vlachopoulos J., Correa C.A. (2009). Rheology and Processing of HDPE/Wood Flour Composites. Int. Polym. Process..

[51] Yang H.S. (2017). Thermal and Dynamic Mechanical Thermal Analysis of Lignocellulosic Material-Filled Polyethylene Biocomposites. J. Therm. Anal. Calorim..

[52] Yang H.S., Kim H.J., Park H.J., Lee B.J., Hwang T.S. (2006). Water Absorption Behaviour and Mechanical Properties of Lignocellulosic Filler-Polyolefin Biocomposites. Compos. Struct..

[53] Balint T., Chang B.P., Mohanty A.K., Misra M. (2020). Underutilized Agricultural Co-Product As A Sustainable Biofiller for Polyamide6, 6: Effect of Carbonization Temperature. Molecules.

[54] Codou A., Guigo N., van Berkel J.G., de Jong E., Sbirrazzuoli N. (2017). Preparation and Crystallization Behavior of Poly(Ethylene 2,5-Furandicarboxylate)/Cellulose Composites by Twin Screw Extrusion. Carbohydr. Polym..

[55] Kaseem M., Hamad K., Deri F., Ko Y.G. (2015). Material Properties of Polyethylene/Wood Composites: A Review of Recent Works. Polym. Sci. Ser. A.

[56] Andrzejewski J., Barczewski M., Szostak M. (2019). Injection Moulding of Highly Filled Polypropylene-Based Biocomposites. Buckwheat Husk and Wood Flour Filler: A Comparison of Agricultural and Wood Industry Waste Utilization. Polymers.

[57] Yang H.S., Wolcott M.P., Kim H.S., Kim H.J. (2005). Thermal Properties of Lignocellulosic Filler-Thermoplastic Polymer Biocomposites. J. Therm. Anal. Calorim..

[58] Väisänen T., Haapala A., Lappalainen R., Tomppo L. (2016). Utilization of Agricultural and Forest Industry Waste and Residues in Natural Fiber-Polymer Composites: A Review. Waste Manag..

[59] Lin N., Huang J., Dufresne A. (2012). Preparation, Properties and Applications of Polysaccharide Nanocrystals in Advanced Functional Nanomaterials: A Review. Nanoscale.

[60] Maneesh T., Singh V.K., Gope P.C., Chaudhary A.K. (2012). Evaluation of Mechanical Properties of Bagasse-Glass Fibre Reinforced Composites. J. Mater. Environ. Sci..

[61] Abba H.A., Zahari I.N., Sapuan S.M., Leman Z. (2017). Characterization of Millet (Pennisetum Glaucum) Husk Fiber (MHF) and Its Use As Filler for High Density Polyethylene (HDPE) Composites. Bioresources.

[62] Arifuzzaman Khan G.M., Alam Shams M.S., Kabir M.R., Gafur M.A., Terano M., Alam M.S. (2013). Influence of Chemical Treatment on the Properties of Banana Stem Fiber and Banana Stem Fiber/Coir Hybrid Fiber Reinforced Maleic Anhydride Grafted Polypropylene/Low-Density Polyethylene Composites. J.Appl. Polym. Sci..

[63] Essabir H., Raji M., Laaziz S.A., Rodrique D., Bouhfid R., Qaiss A. (2018). Thermo-Mechanical Performances of Polypropylene Biocomposites Based on Untreated, Treated and Compatybilized Spent Coffee Grounds. Compos. Part B Eng..

[64] Arrakhiz F.Z., El Achaby M., Bouhfid R., Vaudreuil S., Essassi M., Qaiss A. (2012). Mechanical and Thermal Properties of Polypropylene Reinforced with Alfa Fiber under Different Chemical Treatment. Mater. Des..

[65] Kaewkuk S., Sutapun W., Jarukumjorn K. (2013). Effects of Interfacial Modification and Fiber Content on Physical Properties of Sisal Fiber/Polypropylene Composites. Compos. Part B Eng..

[66] Menezes A.J., Siqueira G., Curvelo A.A.S., Dufresne A. (2009). Extrusion and Characterization of Functionalized Cellulose Whiskers Reinforced Polyethylene Nanocomposites. Polymer.

[67] Kuciel S., Jakubowska P., Kuźniar P. (2014). A Study on the Mechanical Properties and the Influence of Water Uptake and Temperature on Biocomposites Based on Polyethylene from Renewable Sources. Compos. Part B Eng..

[68] Sobczak L., Brüggermann O., Putz R.F. (2013). Polyolefin Composites with Natural Fibres and Wood-Modification of the Fiber/Filler-Matrix Interactions. J. Appl. Polym. Sci..

[69] Yu T., Sun F., Lu M., Li Y. (2018). Water Absorption and Hygrothermal Aging Behavior of Short Ramie Fiber-Reinforced Poly (Lactic Acid) Composites. Polym. Compos..

[70] Sasimowski E., Majewski Ł., Grochowicz M. (2019). Influence of the Design Solutions of Extruder Screw Mixing Tip on Selected Properties of Wheat Bran-Polyethylene Biocomposite. Polymers.

[71] Yeh S.K., Gupta R.K. (2008). Improved Wood-Plastic Composites Through Better Processing. Compos. Part A Appl. Sci. Manuf..

[72] Fernandes E.M., Correlo V.M., Mano J.F., Reis R.L. (2014). Polypropylene-Based Cork-Polymer Composites: Processing Parameters and Properties. Compos. Part B Eng..

[73] Tazi M., Erchiqui F., Godard F., Kaddami H., Ajji A. (2014). Characterization of Rheological and Thermophysical Properties of HDPE-Wood Composite. J. Appl. Polym. Sci..

[74] Li H., Law S., Sain M. (2004). Process Rheology and Mechanical Property Correlationship of Wood Flour-Polypropylene Composites. J. Reinf. Plast. Compos..

[75] Dittenber D.B., GangaRao H.V.S. (2012). Critical Review of Recent Publications on Use of Natural Composites in Infrastructure. Compos. Part A Appl. Sci. Manuf..

[76] ISO 294-1:2017 (2017). Plastics—Injection Moulding of Test Specimens of Thermoplastic Materials—Part 1: General Principles, and Moulding of Multipurpose and Bar Test Specimens.

[77] Dowlex 2631.10EU Polyethylene Resin—Technical Data Sheet. https://www.dow.com/en-us/document-viewer.html?ramdomVar=9174234036439704424&docPath=/content/dam/dcc/ocuments/en-us/productdatasheet/400/400-00089061en-dowlex-2631ue-tds.pdf.

[78] Stevenson L., Philips F., O’Sullivan K., Walton J. (2012). Wheat Bran: Its Composition and Benefits to Health, A European Perspective. Int. J. Food Sci. Nutr..

[79] ASTM E308 (2018). Standard Practice for Computing the Colour of Objects by Using the CIE System.

[80] ISO 2813:2001 (2001). Paints and Varnishes—Determination of Gloss Value at 20 Degrees, 60 Degrees and 85 Degrees.

[81] ISO 294-4:2001 (2001). Plastics—Injection Moulding of Test Specimens of Thermoplastic Materials—Part 4: Determination of Moulding Shrinkage.

[82] ISO 527-2:2012 (2012). Plastics—Determination of Tensile Properties—Part 2: Test Conditions for Moulding and Extrusion Plastics.

[83] ISO 1133-1:2011 (2011). Plastics—Determination of the Melt Mass-Flow Rate (MFR) and Melt Volume-Flow Rate (MVR) of Thermoplastics—Part 1: Standard Method.

[84] ISO 17744:2004 (2004). Plastics—Determination of Specific Volume as A Function of Temperature and Pressure (pvT Diagram)—Piston Apparatus Method.

[85] ISO 11357-1:2016 (2016). Plastics—Differential Scanning Calorimetry (DSC)—Part 1: General Principles.

[86] Mathot V.B.F. (1994). Calorimetry and Thermal Analysis of Polymers.

[87] Montanes N., Quiles-Carrillo L., Ferrandiz S., Fenollar O., Boronat T. (2019). Effects of Lignocellulosic Fillers from Waste Thyme on Melt Flow Behavior and Processability of Wood Plastic Composites (WPC) with Biobased Poly (Ethylene) by Injection Moulding. J. Polym. Environ..

[88] Orji B.O., McDonald A.G. (2020). Evaluation of the Mechanical, Thermal and Rheological Properties of Recycled Polyolefins Rice-Hull Composites. Materials.

[89] Teuber L., Militz H., Krause A. (2015). Processing of Wood Plastic Composites: The Influence of Feeding Method and Polymer Melt Flow Rate on Particle Degradation. J. Appl. Polym. Sci..

[90] Pozo C., Rodríguez-Llamazares S., Bouza R., Barral L., Castaño J., Müller N. (2018). Study of the Structural Order of Native Starch Granules Using Combined FTIR and XRD Analysis. J. Polym. Res..

[91] Szymańska-Chargot M., Zdunek A. (2013). Use of FT-IR Spectra and PCA to the Bulk Characterization of Cell Wall Residues of Fruits and Vegetables along the Fraction Process. Food Biophys..

[92] Piotrowicz I.B.B., Salas-Mellado M.M. (2017). Protein Concentrates from Defatted Rice Bran: Preparation and Characterization. Food Sci. Technol..

[93] Poletto M., Zattera A.J., Santana R.M.C. (2012). Structural Differences between Wood Species: Evidence from Chemical Composition, FTIR Spectroscopy, and Thermogravimetric Analysis. J. Appl. Polym. Sci..

[94] Liu W., Wang Y.J., Sun Z. (2003). Effects of Polyethylene-Grafted Maleic Anhydride (PE-g-MA) on Thermal Properties, Morphology, and Tensile Properties of Low Density Polyethylene (LDPE) and Corn Starch Blends. J. Appl. Polym. Sci..

[95] Pabiot J., Verdu J. (1981). The Change in Mechanical Behavior of Linear Polymers during Photochemical Aging. Polym. Eng. Sci..

[96] Sikora J.W., Gajdoš I., Puszka A. (2019). Polyethylene-Matrix Composites with Halloysite Nanotubes with Enhanced Physical/Thermal Properties. Polymers.

[97] Shahzad A., Isaac D.H., Thakur V.K. (2014). Weathering of lignocellulosic polymer composites. Lignocellulosic Polymer Composites. Processing, Characterisation and Properties.

[98] Ashori A. (2008). Wood-Plastic Composites as Promising Green-Composites for Automotive Industries. Bioresour. Technol..

[99] Barczewski M., Mysiukiewicz O., Kloziński A. (2018). Complex Modification Effect of linseed cake as an Agricultural Waste Filler Used in High Density Polyethylene Composites. Iran. Polym. J..

[100] Zhang Y., Zhang S.Y., Choi P. (2008). Effects of Wood Fiber Content and Coupling Agent Content on Tensile Properties of Wood Fiber Polyethylene Composites. Holz Roh Werkst.

